# Profile of Participants and Genotype Distributions of 108 Polymorphisms in a Cross-Sectional Study of Associations of Genotypes With Lifestyle and Clinical Factors: A Project in the Japan Multi-Institutional Collaborative Cohort (J-MICC) Study

**DOI:** 10.2188/jea.JE20100139

**Published:** 2011-05-05

**Authors:** Kenji Wakai, Nobuyuki Hamajima, Rieko Okada, Mariko Naito, Emi Morita, Asahi Hishida, Sayo Kawai, Kazuko Nishio, Guang Yin, Yatami Asai, Keitaro Matsuo, Satoyo Hosono, Hidemi Ito, Miki Watanabe, Takakazu Kawase, Takeshi Suzuki, Kazuo Tajima, Keitaro Tanaka, Yasuki Higaki, Megumi Hara, Takeshi Imaizumi, Naoto Taguchi, Kazuyo Nakamura, Hinako Nanri, Tatsuhiko Sakamoto, Mikako Horita, Koichi Shinchi, Yoshikuni Kita, Tanvir Chowdhury Turin, Nahid Rumana, Kenji Matsui, Katsuyuki Miura, Hirotsugu Ueshima, Naoyuki Takashima, Yasuyuki Nakamura, Sadao Suzuki, Ryosuke Ando, Akihiro Hosono, Nahomi Imaeda, Kiyoshi Shibata, Chiho Goto, Nami Hattori, Mitsuru Fukatsu, Tamaki Yamada, Shinkan Tokudome, Toshiro Takezaki, Hideshi Niimura, Kazuyo Hirasada, Akihiko Nakamura, Masaya Tatebo, Shin Ogawa, Noriko Tsunematsu, Shirabe Chiba, Haruo Mikami, Suminori Kono, Keizo Ohnaka, Ryoichi Takayanagi, Yoshiyuki Watanabe, Etsuko Ozaki, Masako Shigeta, Nagato Kuriyama, Aya Yoshikawa, Daisuke Matsui, Isao Watanabe, Kaoru Inoue, Kotaro Ozasa, Satoko Mitani, Kokichi Arisawa, Hirokazu Uemura, Mineyoshi Hiyoshi, Hidenobu Takami, Miwa Yamaguchi, Mariko Nakamoto, Hideo Takeda, Michiaki Kubo, Hideo Tanaka

**Affiliations:** 1 Department of Preventive Medicine, Nagoya University Graduate School of Medicine, Nagoya, Japan; 2 Seirei Social Welfare Community, Hamamatsu, Japan; 3 Division of Epidemiology and Prevention, Aichi Cancer Center Research Institute, Nagoya, Japan; 4 Department of Medical Oncology and Immunology, Nagoya City University Graduate School of Medical Science, Nagoya, Japan; 5 Aichi Cancer Center Research Institute, Nagoya, Japan; 6 Department of Preventive Medicine, Faculty of Medicine, Saga University, Saga, Japan; 7 Laboratory of Exercise Physiology, Faculty of Sports and Health Science, Fukuoka University, Fukuoka, Japan; 8 Asakura Health Welfare Environment Office, Fukuoka Prefectural Government, Asakura, Japan; 9 Division of International Health and Nursing, Faculty of Medicine, Saga University, Saga, Japan; 10 Department of Health Science, Shiga University of Medical Science, Otsu, Japan; 11 Lifestyle-Related Disease Prevention Center, Shiga University of Medical Science, Otsu, Japan; 12 Department of Cardiovascular Epidemiology, Kyoto Women’s University, Kyoto, Japan; 13 Department of Public Health, Nagoya City University Graduate School of Medical Sciences, Nagoya, Japan; 14 Public Health Center, Okazaki City Medical Association, Okazaki, Japan; 15 Department of International Island and Community Medicine, Kagoshima University Graduate School of Medical and Dental Science, Kagoshima, Japan; 16 Division of Cancer Registry, Prevention and Epidemiology, Chiba Cancer Center, Chiba, Japan; 17 Department of Preventive Medicine, Graduate School of Medical Sciences, Kyushu University, Fukuoka, Japan; 18 Department of Geriatric Medicine, Graduate School of Medical Sciences, Kyushu University, Fukuoka, Japan; 19 Department of Medicine and Bioregulatory Science, Graduate School of Medical Sciences, Kyushu University, Fukuoka, Japan; 20 Department of Epidemiology for Community Health and Medicine, Kyoto Prefectural University of Medicine, Kyoto, Japan; 21 Unit for Liveable Cities, Graduate School of Medicine, Kyoto University, Kyoto, Japan; 22 Department of Preventive Medicine, Institute of Health Biosciences, The University of Tokushima Graduate School, Tokushima, Japan; 23 Laboratory for Genotyping Development, Center for Genomic Medicine, RIKEN, Yokohama, Japan

**Keywords:** allele frequency, cross-sectional studies, gene–environment interactions, Japan Multi-institutional Collaborative Cohort Study, polymorphism

## Abstract

**Background:**

Most diseases are thought to arise from interactions between environmental factors and the host genotype. To detect gene–environment interactions in the development of lifestyle-related diseases, and especially cancer, the Japan Multi-institutional Collaborative Cohort (J-MICC) Study was launched in 2005.

**Methods:**

We initiated a cross-sectional study to examine associations of genotypes with lifestyle and clinical factors, as assessed by questionnaires and medical examinations. The 4519 subjects were selected from among participants in the J-MICC Study in 10 areas throughout Japan. In total, 108 polymorphisms were chosen and genotyped using the Invader assay.

**Results:**

The study group comprised 2124 men and 2395 women with a mean age of 55.8 ± 8.9 years (range, 35–69 years) at baseline. Among the 108 polymorphisms examined, 4 were not polymorphic in our study population. Among the remaining 104 polymorphisms, most variations were common (minor allele frequency ≥0.05 for 96 polymorphisms). The allele frequencies in this population were comparable with those in the HapMap-JPT data set for 45 Japanese from Tokyo. Only 5 of 88 polymorphisms showed allele-frequency differences greater than 0.1. Of the 108 polymorphisms, 32 showed a highly significant difference in minor allele frequency among the study areas (*P* < 0.001).

**Conclusions:**

This comprehensive data collection on lifestyle and clinical factors will be useful for elucidating gene–environment interactions. In addition, it is likely to be an informative reference tool, as free access to genotype data for a large Japanese population is not readily available.

## INTRODUCTION

Although the etiology of many diseases is not completely understood, most are likely to be caused by interactions between hazardous environmental factors and the host genome. Recent advances in genotyping techniques have allowed many epidemiologic studies to investigate gene–environment interactions in chronic diseases.^1^^–^^4^ Cohort and case–control studies focusing on such interactions are ongoing worldwide, and these investigations use DNA from established and new cohorts.^5^^–^^8^ Understanding gene–environment interactions requires long-term cohort studies to clarify the temporality of associations and to avoid information and selection biases that are inevitable in cross-sectional and case–control studies.^9^ For most multifactorial diseases, such cohort studies must be conducted on a large scale to ensure significant results.

The Japan Multi-institutional Collaborative Cohort (J-MICC) Study is a new cohort study that was launched in 2005 to examine gene–environment interactions in lifestyle-related diseases, especially cancers. It is supported by a research grant for Scientific Research on Special Priority Areas of Cancer from the Japanese Ministry of Education, Culture, Sports, Science and Technology (MEXT).^10^^,^^11^ The J-MICC Study group is composed of 10 cohorts surveyed by 10 independent research teams.^12^^,^^13^

Although the long-term aim of this study is to elucidate gene–environment interactions in the whole cohort, some of its research objectives will be achieved by cross-sectional studies. In 2009, we started a cross-sectional study to examine correlations between lifestyle and medical factors, as assessed using questionnaires and medical examinations, and the distribution of possible related genotypes. Here we describe the recruitment and profile of the participants, including their genotype analysis, and selected demographic, lifestyle, and medical characteristics.

## METHODS

### Study participants, data collection, and blood sampling

The participants in this study completed questionnaires on lifestyle factors and diseases and donated blood samples at the time of the baseline survey for the J-MICC Study. The details of the J-MICC Study have been described elsewhere.^10^ The participants were enrolled in 9 study areas throughout Japan between 2005 and 2008, and in 1 area in 2004, under the supervision of an associate member of the J-MICC Study. The study participants were enrolled from the community by mailing invitation letters or distributing leaflets (3 areas), or by recruiting patients at their first visit to a cancer hospital (1 area) or at health checkups (6 areas). The response rates for the baseline survey were 7.0%, 36.5%, 25.9%, 58.4%, 60.1%, 37.6%, 14.0%, 24.0%, 19.7%, and 65.5% for the Chiba, Shizuoka, Okazaki, Aichi Cancer Center, Takashima, Kyoto, Tokushima, Fukuoka, Saga, and Amami areas, respectively. For cases in which the baseline survey is still ongoing in a cohort, the latest response rate (as of 30 September 2010 or later) was used. Anthropometry, blood pressure, and blood chemistry data obtained from health checkups were available in 8 of the study areas. The subjects for the cross-sectional study comprised 500 to 600 participants enrolled consecutively in each area of the J-MICC Study, except in 2 areas, where fewer participants had been recruited. The recruitment period for the present study, however, was arbitrarily defined by the researchers in each area after the enrollment.

Of the 5108 men and women initially selected, we excluded participants for whom we did not have sufficient DNA (*n* = 442), appropriate informed consent (*n* = 8), questionnaire data (*n* = 9), or local government registration of residence in the study area (*n* = 7), as well as anyone who had declined follow up (*n* = 2) or withdrew from the study (*n* = 1), and the 120 participants who were younger than 35 years or older than 69 years. Thus, our final study group comprised 4519 participants aged 35 to 69 years.

All the participants included in this analysis had provided written informed consent. The ethics committees of Nagoya University School of Medicine (the affiliation of the former principal investigator, Nobuyuki Hamajima) and the other participating institutions approved the protocol for the J-MICC Study.

### Genotyping

We chose 107 single nucleotide polymorphisms (SNPs) and 1 insertion/deletion polymorphism for genotyping, based on their potential relevance to the lifestyle and medical factors described in the next section (“Lifestyle and clinical data”). Researchers from all participating cohorts proposed potentially relevant polymorphisms, and those selected for inclusion in the present study were determined through discussion among the members of the J-MICC Study Group.

In all study areas except Fukuoka, buffy coat fractions were prepared from blood samples and stored at −80°C at the central J-MICC Study office. DNA was extracted from all buffy coat fractions using a BioRobot M48 Workstation (Qiagen Group, Tokyo, Japan) at the central study office. For the samples from the Fukuoka area, DNA was extracted locally from samples of whole blood, using an automatic nucleic acid isolation system (NA-3000, Kurabo, Co., Ltd, Osaka, Japan). The buffy coat fractions or DNA samples were anonymized in a linkable manner^14^ and then sent to the central office.

The selected polymorphisms were genotyped using the multiplex polymerase chain reaction (PCR)-based Invader assay^15^ (Third Wave Technologies, Madison, WI, USA) at the Laboratory for Genotyping Development, Center for Genomic Medicine, RIKEN.

### Lifestyle and clinical characteristics

The lifestyle factors considered were smoking and drinking habits, coffee consumption, sleep, and mental stress, while the clinical characteristics were height, weight, blood pressure, blood glucose, glycated hemoglobin (HbA1c), serum triglyceride, total and high-density lipoprotein (HDL) cholesterol, uric acid, aspartate aminotransferase (AST), alanine aminotransferase (ALT), gamma-glutamyltransferase (γ-GT), C-reactive protein (CRP), creatinine, and bone mineral density. Ages at menarche and menopause were also ascertained.

We used a standard questionnaire in all study areas except the Fukuoka area, where some questions are slightly different from those of other areas. Furthermore, a validated food-frequency questionnaire was used for the dietary assessment.^16^^–^^19^ We were unable to directly control the quality of information from health examinations because most data were obtained at routine health checkups offered by other institutions. However, the J-MICC Study Group is now collecting information on participation in the Japan Medical Association’s quality control program for clinical laboratories and the instruction manuals used for measurement of blood pressure, height, and weight. For the current report, participants whose blood was drawn less than 3 hours after their last meal were excluded from the analysis of serum lipids and blood glucose.

### Statistical analysis

We tabulated selected baseline characteristics by sex and 10-year age group or by sex and study area. In this analysis, body mass index (BMI; kg/m^2^) was calculated on the basis of self-reported height and body weight, as independent measurements were not available in some study areas. In the case of educational attainment, participants from the Fukuoka area were excluded from the analysis because the questionnaire used there had not included this item. Participants who consumed alcohol at least once a week were classified as drinkers. To compare characteristics among participating cohorts, we attempted to adjust for age by using the direct method (for proportions) or the general linear model (for means). The variations among study areas, however, were not significantly altered after adjusting for age. Thus, in this report, we present only crude figures by sex and study area. The difference in the minor allele frequency (MAF) among the cohorts was tested by the chi-square test for contingency tables. The MAF of the *ABCC11* Arg180Gly (T/C) polymorphism by study area is not presented here because the inter-area variation in the distribution of this genotype will be reported in a separate article.

Genotypes with distributions that departed from the Hardy–Weinberg equilibrium were assessed using the exact test^20^ with the genhwi command of Stata version 8.0 (Stata Corp, College Station, TX, USA). Other statistical analyses were performed using Statistical Analysis System version 9.1 (SAS Institute Inc, Cary, NC, USA).^21^ To compare the allele frequencies of genotypes in our study with those in another Japanese population, we used data from HapMap, which is an open access database that includes allele frequencies for 45 Japanese in Tokyo (HapMap-JPT, http://www.ncbi.nlm.nih.gov/snp). Of the 108 polymorphisms of interest, we made comparisons for 88. The 20 polymorphisms excluded from our analysis showed no minor alleles in our study group (*n* = 4), were not represented (*n* = 15), or had invalid data (*n* = 1, 100% heterozygotes) in the HapMap-JPT data set.

## RESULTS

Our analysis included 2124 men (47.0%) and 2395 women (53.0%) with a mean age ± standard deviation at baseline of 55.8 ± 8.9 years (range, 35–69 years). There were considerable differences in the age and sex distributions of different study areas (Table 1). In Fukuoka and Saga, the participants originally enrolled in the J-MICC Study were limited to adults aged 50 years or older and 40 years or older, respectively.

**Table 1.** Sex and age distribution of study participants by study area

Study area	Men										Women									
	
	Age (years)								Total		Age (years)								Total	
	
	35–39		40–49		50–59		60–69				35–39		40–49		50–59		60–69			
									
	*n*	*%*	*n*	*%*	*n*	*%*	*n*	*%*	*n*	*%*	*n*	*%*	*n*	*%*	*n*	*%*	*n*	*%*	*n*	*%*
Chiba	4	2.7	22	14.8	56	37.6	67	45.0	149	100.0	30	8.4	138	38.7	118	33.1	71	19.9	357	100.0
Shizuoka	21	5.0	122	29.3	175	42.1	98	23.6	416	100.0	16	10.1	35	22.0	70	44.0	38	23.9	159	100.0
Okazaki	13	4.8	29	10.6	66	24.2	165	60.4	273	100.0	12	4.7	45	17.6	85	33.3	113	44.3	255	100.0
Aichi Cancer Center	12	4.4	32	11.6	115	41.8	116	42.2	275	100.0	33	10.9	88	29.0	102	33.7	80	26.4	303	100.0
Takashima	7	4.2	18	10.7	45	26.8	98	58.3	168	100.0	27	7.2	59	15.8	102	27.3	186	49.7	374	100.0
Kyoto	37	30.3	31	25.4	48	39.3	6	4.9	122	100.0	9	23.7	19	50.0	9	23.7	1	2.6	38	100.0
Tokushima	8	11.0	21	28.8	24	32.9	20	27.4	73	100.0	1	4.5	9	40.9	10	45.5	2	9.1	22	100.0
Fukuoka	0	0.0	0	0.0	60	31.9	128	68.1	188	100.0	0	0.0	0	0.0	96	37.5	160	62.5	256	100.0
Saga	0	0.0	31	12.7	82	33.5	132	53.9	245	100.0	0	0.0	64	19.3	127	38.4	140	42.3	331	100.0
Amami	1	0.5	53	24.7	82	38.1	79	36.7	215	100.0	1	0.3	77	25.7	135	45.0	87	29.0	300	100.0

Total	103	4.8	359	16.9	753	35.5	909	42.8	2124	100.0	129	5.4	534	22.3	854	35.7	878	36.7	2395	100.0

Table 2 summarizes selected demographic, lifestyle, and medical characteristics of the participants by sex and age. Within our sample, 29.1% of men and 7.1% of women were current smokers. More than two thirds (71.4%) of men drank alcoholic beverages at least once a week, as did 27.7% of the women. Table 3 presents data on selected lifestyle and medical variables of the participants by sex and study area. Considerable variations were found among the participating cohorts.

**Table 2.** Selected demographic, lifestyle, and medical characteristics of participants by sex and age

	Men					Women				
		
	Age (years)				Total	Age (years)				Total
		
	35–39	40–49	50–59	60–69		35–39	40–49	50–59	60–69	
*n*	103	359	753	909	2124	129	534	854	878	2395
Educational attainment (%)^a^										
Elementary/junior ​ high school	1.0	3.1	10.0	20.9	12.6	2.3	2.1	9.8	31.6	14.7
High school	42.7	37.8	41.9	43.7	41.9	41.9	38.4	50.7	46.4	45.6
Vocational school	14.6	9.5	6.8	3.2	6.3	15.5	13.9	12.7	10.6	12.5
Junior college	3.9	4.8	3.9	2.2	3.4	24.8	26.5	16.1	7.4	16.3
University	33.0	39.5	34.8	27.6	32.7	15.5	17.7	10.2	3.8	10.2
Postgraduate school	4.9	5.0	2.2	1.7	2.6	0.0	1.5	0.5	0.0	0.6
Others	0.0	0.3	0.4	0.7	0.5	0.0	0.0	0.0	0.1	0.0
Current smokers (%)	39.8	32.3	34.8	21.9	29.1	11.6	10.3	7.4	4.1	7.1
Ex-smokers (%)	25.2	35.7	41.8	48.6	42.9	5.4	7.9	4.8	2.8	4.8
Current drinkers (%)^b^	60.2	70.8	75.1	69.9	71.4	32.6	34.6	27.5	22.8	27.7
Exercise ≥1/month (%)	75.7	81.6	79.7	86.8	82.9	62.0	70.4	74.9	82.5	76.0
Body mass ​index ≥25.0 (%)	37.9	32.2	32.6	24.9	29.5	9.5	16.5	20.0	21.8	19.3
History of disease (%)										
Diabetes	1.9	4.5	8.4	13.9	9.7	0.0	0.6	3.4	7.1	3.8
Hypertension	3.9	8.9	23.2	36.2	25.2	0.0	4.3	17.1	30.5	17.9
Coronary heart ​ disease	0.0	1.1	2.1	7.3	4.0	0.8	0.6	2.8	5.3	3.0
Stroke	0.0	1.1	2.7	3.9	2.8	2.3	0.9	1.6	2.9	2.0
Cancer	6.1	2.0	7.7	12.6	8.6	2.8	5.0	9.3	6.4	6.9
Blood pressure and blood chemistry^c^										
Systolic blood ​ pressure (mm Hg)	120.4 ± 14.3	121.1 ± 15.4	129.7 ± 17.9	135.4 ± 19.3	130.1 ± 18.8	109.6 ± 11.4	116.8 ± 18.3	125.0 ± 18.9	133.4 ± 19.0	126.5 ± 19.9
Diastolic blood ​ pressure (mm Hg)	73.6 ± 10.3	77.6 ± 11.7	81.9 ± 12.6	82.1 ± 11.1	80.8 ± 11.9	65.4 ± 7.4	72.0 ± 11.9	76.7 ± 11.6	79.0 ± 10.8	76.4 ± 11.7
Total cholesterol ​ (mg/dl)	191.3 ± 28.9	205.3 ± 30.2	206.6 ± 32.5	205.2 ± 33.1	205.2 ± 32.3	185.6 ± 25.2	202.4 ± 31.0	223.3 ± 34.2	223.1 ± 33.5	217.9 ± 34.6
HDL-cholesterol ​ (mg/dl)	56.2 ± 16.0	57.6 ± 15.4	59.1 ± 15.8	59.7 ± 16.2	58.9 ± 15.9	73.1 ± 15.7	68.7 ± 15.3	68.9 ± 15.0	66.2 ± 16.0	68.0 ± 15.5
Triglyceride (mg/dl)	128.6 ± 74.9	146.5 ± 96.5	135.1 ± 93.0	132.0 ± 92.9	135.9 ± 93.0	64.8 ± 28.4	88.0 ± 61.2	104.9 ± 64.2	113.7 ± 69.2	103.4 ± 65.7
Blood glucose (mg/dl)	98.0 ± 27.2	98.3 ± 15.7	103.2 ± 17.6	103.9 ± 23.3	102.1 ± 20.3	86.8 ± 7.1	92.4 ± 19.7	95.9 ± 16.8	97.1 ± 15.8	95.1 ± 16.9
HbA1c (%)	4.96 ± 0.32	5.12 ± 0.48	5.32 ± 0.86	5.30 ± 0.75	5.27 ± 0.74	4.86 ± 0.27	4.96 ± 0.36	5.17 ± 0.64	5.26 ± 0.57	5.17 ± 0.57

Plus-minus values are means ± SDs.

^a^Participants in Fukuoka area were excluded from the analysis because they were not asked about educational attainment in the questionnaire.

^b^Individuals who drank alcoholic beverages ≥1 day/week.

^c^Not available in some study areas, as shown in Table 3.

**Table 3.** Selected lifestyle and medical characteristics of participants by sex and study area

	*n*	Study area										
	
		Total	Chiba	Shizuoka	Okazaki	ACC	Takashima	Kyoto	Tokushima	Fukuoka	Saga	Amami
Men												
Current smokers (%)	2123	29.1	23.5	23.6	25.6	31.0	35.7	41.8	31.5	33.0	34.3	23.3
Ex-smokers (%)	2123	42.9	42.3	48.3	50.2	44.2	33.3	33.6	38.4	39.4	42.9	39.1
Current drinkers (%)^a^	2121	71.4	78.5	73.4	62.6	66.9	73.8	68.0	61.6	70.2	71.4	84.1
Exercise ≥1/month (%)	2109	82.9	87.1	88.7	81.0	83.5	60.1	81.0	94.5	89.9	85.3	75.9
Body mass index ≥25.0 (%)	2114	29.5	29.1	25.2	26.4	22.6	27.0	23.8	41.1	31.4	27.7	52.8
History of hypertension (%)	2052	25.2	28.2	14.7	29.5	24.0	28.7	9.9	21.9	44.4	28.6	32.1

Systolic blood pressure (mm Hg)	1698	130.1 ± 18.8	NA	121.9 ± 14.8	128.7 ± 15.2	NA	133.0 ± 19.5	122.8 ± 16.0	119.8 ± 17.6	144.0 ± 19.6	138.3 ± 19.3	132.0 ± 17.7
Diastolic blood pressure ​ (mm Hg)	1698	80.8 ± 11.9	NA	76.5 ± 10.5	81.4 ± 9.1	NA	81.2 ± 11.2	74.3 ± 11.9	73.7 ± 12.8	88.0 ± 11.1	86.0 ± 12.8	81.9 ± 11.1
Total cholesterol (mg/dl)	1296	205.2 ± 32.3	NA	201.3 ± 29.3	205.9 ± 32.0	NA	208.3 ± 35.6	—^b^	209.0 ± 31.4	209.9 ± 31.5	203.6 ± 33.6	208.0 ± 35.7
HDL-cholesterol (mg/dl)	1378	58.9 ± 15.9	NA	58.3 ± 15.4	64.1 ± 17.5	NA	59.4 ± 17.0	61.4 ± 17.6	52.5 ± 11.6	54.5 ± 16.1	55.3 ± 14.2	57.7 ± 13.4
Triglyceride (mg/dl)	1377	135.9 ± 93.0	NA	122.8 ± 68.6	120.8 ± 86.5	NA	116.9 ± 60.0	127.9 ± 85.7	126.7 ± 64.2	181.5 ± 131.9	164.9 ± 93.6	165.7 ± 130.8
Blood glucose (mg/dl)	1175	102.1 ± 20.3	NA	101.1 ± 14.3	100.0 ± 17.0	NA	97.9 ± 16.3	98.7 ± 25.0	104.2 ± 26.4	NA	NA	110.3 ± 28.8
HbA1c (%)	1354	5.27 ± 0.74	NA	5.27 ± 0.56	5.36 ± 0.64	NA	5.24 ± 0.79	NA	5.17 ± 0.52	5.17 ± 0.82	5.28 ± 1.04	5.31 ± 0.42

Women												
Current smokers (%)	2390	7.1	7.6	5.0	6.3	12.4	5.6	15.8	0.0	9.0	5.4	4.4
Ex-smokers (%)	2390	4.8	6.7	4.4	5.9	8.4	1.3	7.9	4.6	4.7	5.7	1.0
Current drinkers (%)^a^	2383	27.7	36.1	27.2	22.4	31.7	24.6	34.2	22.7	25.0	28.2	23.3
Exercise ≥1/month (%)	2381	76.0	82.8	81.8	83.9	70.9	55.6	44.7	81.8	88.3	83.4	73.1
Body mass index ≥25.0 (%)	2359	19.3	12.8	15.5	17.3	14.0	21.9	15.8	27.3	19.5	16.5	36.2
History of hypertension (%)	2289	17.9	10.4	15.8	19.3	12.9	18.5	7.9	18.2	37.9	17.6	22.0

Systolic blood pressure (mm Hg)	1732	126.5 ± 19.9	NA	115.7 ± 16.8	122.7 ± 15.6	NA	124.6 ± 19.5	120.4 ± 19.5	111.5 ± 11.7	137.5 ± 21.9	129.5 ± 19.5	126.8 ± 19.0
Diastolic blood pressure ​ (mm Hg)	1732	76.4 ± 11.7	NA	70.3 ± 10.5	76.7 ± 9.5	NA	73.6 ± 11.4	71.8 ± 11.1	68.2 ± 10.3	84.0 ± 11.3	77.9 ± 12.2	76.0 ± 10.4
Total cholesterol (mg/dl)	1318	217.9 ± 34.6	NA	206.4 ± 31.9	213.5 ± 34.1	NA	222.6 ± 36.5	NA	210.0 ± 35.7	230.1 ± 31.4	220.3 ± 33.5	218.0 ± 33.7
HDL-cholesterol (mg/dl)	1347	68.0 ± 15.5	NA	72.3 ± 16.1	75.0 ± 16.9	NA	67.0 ± 15.0	69.3 ± 13.7	65.5 ± 12.0	66.1 ± 16.6	64.3 ± 14.1	63.4 ± 12.6
Triglyceride (mg/dl)	1347	103.4 ± 65.7	NA	80.0 ± 40.9	97.3 ± 60.9	NA	96.8 ± 54.9	68.6 ± 27.8	84.3 ± 41.2	128.0 ± 66.0	129.2 ± 83.4	109.4 ± 74.2
Blood glucose (mg/dl)	1072	95.1 ± 16.9	NA	91.9 ± 8.3	94.7 ± 13.7	NA	91.0 ± 10.2	89.6 ± 7.1	101.9 ± 31.9	NA	NA	101.8 ± 24.3
HbA1c (%)	1396	5.17 ± 0.57	NA	5.15 ± 0.35	5.32 ± 0.57	NA	5.11 ± 0.54	NA	—^b^	5.16 ± 0.65	5.10 ± 0.59	—^b^

Abbreviations: ACC, Aichi Cancer Center; HDL, high-density lipoprotein; NA, not available.

Plus-minus values are means ± SDs.

^a^Individuals who drank alcoholic beverages ≥1 day/week.

^b^Data available for fewer than 20 participants.

The genotype distributions and allele frequencies of the analyzed genetic polymorphisms are summarized in Table 4. The call rate ranged from 99.40% to 99.98%. Of the 108 polymorphisms, the 4 SNPs for which we found no different alleles were *APOA1* Arg184Pro (G/C), *ESR1* IVS1-351A/G (*Xba* I), *LCAT/SLC12A4* Ser232Thr (T/A), and *SCARB1* Val135Ile (G/A). For the remaining 104 polymorphisms, the MAF varied from 0.016 (*PTGS2*(*COX2*) C-163G) to 0.492 (*CETP* Ile405Val (A/G)), and most of the variations were common (MAF ≥0.05 for 96 polymorphisms).

**Table 4.** Genotype distributions and allele frequencies of 108 selected genetic polymorphisms (107 SNPs and 1 insertion/deletion polymorphism)

Gene	Polymorphism	rs number	Genotype^a^			*n*^a^				Frequency (proportion)						*P* for HWE	MAF

										Observed			Expected^b^				
			
			AA	Aa	aa	AA	Aa	aa	XX	AA	Aa	aa	AA	Aa	aa		
*ABCA1*	C-565T	rs2422493	CC	CT	TT	1634	2137	746	2	0.362	0.473	0.165	0.358	0.481	0.161	0.29	0.402
*ABCA1*	G-191C	rs1800976	GG	GC	CC	1628	2144	746	1	0.360	0.475	0.165	0.357	0.481	0.162	0.37	0.402
*ABCA1*	G-17C	rs2740483	GG	GC	CC	2211	1898	409	1	0.489	0.420	0.091	0.489	0.420	0.090	0.94	0.301
*ABCA1*	Val825Ile (G/A)	rs2066715	GG	GA	AA	1877	2064	577	1	0.415	0.457	0.128	0.415	0.459	0.127	0.80	0.356
*ABCA1*	Val771Met (G/A)	rs2066718	GG	GA	AA	3945	550	23	1	0.873	0.122	0.005	0.872	0.123	0.004	0.40	0.066
*ABCA1*	Arg1587Lys (G/A)	rs2230808	GG	GA	AA	1673	2154	689	3	0.370	0.477	0.153	0.371	0.476	0.153	0.95	0.391
*ABCC11*	Arg180Gly (T/C)	rs17822931	TT	TC	CC	3368	1051	95	5	0.746	0.233	0.021	0.744	0.237	0.019	0.23	0.137
*ACE*	Insertion/Deletion (I/D)	rs1799752	I/I	I/D	D/D	1854	2021	634	10	0.411	0.448	0.141	0.404	0.463	0.133	0.029	0.365
*ADD1*	Trp460Gly (T/G)	rs4961	TT	TG	GG	1399	2163	956	1	0.310	0.479	0.212	0.301	0.495	0.203	0.026	0.451
*ADH1B*	His47Arg (A/G)	rs1229984	AA	AG	GG	2607	1659	250	3	0.577	0.367	0.055	0.579	0.364	0.057	0.54	0.239
*ADH1C*	Arg272Gln (C/T)	rs1693482	CC	CT	TT	3882	591	43	3	0.860	0.131	0.010	0.856	0.139	0.006	0.0005	0.075
*ADIPOQ*	C-11377G	rs266729	CC	CG	GG	2553	1663	301	2	0.565	0.368	0.067	0.561	0.376	0.063	0.18	0.251
*ADIPOQ*	Gly15Gly (T/G)	rs2241766	TT	TG	GG	2314	1789	415	1	0.512	0.396	0.092	0.504	0.412	0.084	0.011	0.290
*ADIPOQ*	G276T	rs1501299	GG	GT	TT	2498	1674	346	1	0.553	0.371	0.077	0.545	0.387	0.069	0.006	0.262
*ADIPOQ*	IVS-3971A/G	rs822396	AA	AG	GG	3984	517	16	2	0.882	0.114	0.004	0.882	0.114	0.004	1.00	0.061
*ADIPOR1*	G-8503A	rs6666089	GG	GA	AA	4234	272	7	6	0.938	0.060	0.002	0.938	0.061	0.001	0.22	0.032
*ADIPOR1*	C5843T	rs1342387	CC	CT	TT	1230	2206	1073	10	0.273	0.489	0.238	0.268	0.499	0.233	0.17	0.483
*ADIPOR1*	C10224G	rs7539542	CC	CG	GG	2788	1505	225	1	0.617	0.333	0.050	0.614	0.339	0.047	0.24	0.216
*ADRB2*	Gln27Glu (C/G)	rs1042714	CC	CG	GG	3943	551	20	5	0.874	0.122	0.004	0.873	0.122	0.004	0.81	0.065
*ADRB3*	Trp64Arg (T/C)	rs4994	TT	TC	CC	2932	1397	182	8	0.650	0.310	0.040	0.648	0.314	0.038	0.34	0.195
*AGT*	Thr174Met (C/T)	rs4762	CC	CT	TT	3615	848	52	4	0.801	0.188	0.012	0.800	0.189	0.011	0.75	0.105
*AGT*	Thr235Met (C/T)	rs699	CC	CT	TT	2989	1372	157	1	0.662	0.304	0.035	0.662	0.304	0.035	1.00	0.187
*AGTR1(ATR1)*	A1166C (at 3′UTR)	rs5186	AA	AC	CC	3777	695	45	2	0.836	0.154	0.010	0.834	0.159	0.008	0.048	0.087
*ALDH2*	Glu487Lys (G/A)	rs671	GG	GA	AA	2544	1649	321	5	0.564	0.365	0.071	0.557	0.379	0.064	0.018	0.254
*APOA1*	Ala61Thr (C/T)	rs12718465	CC	CT	TT	4036	463	18	2	0.894	0.103	0.004	0.893	0.104	0.003	0.25	0.055
*APOA1*	Arg184Pro (G/C)	rs5078	GG	GC	CC	4512	0	0	7	1.000	0.000	0.000	1.000	0.000	0.000	1.00	0.000
*APOA5*	T-1131C	rs662799	TT	TC	CC	1955	2001	556	7	0.433	0.443	0.123	0.429	0.452	0.119	0.21	0.345
*APOA5*	Gly185Cys (G553T)	rs2075291	GG	GT	TT	3920	559	32	8	0.869	0.124	0.007	0.867	0.129	0.005	0.020	0.069
*APOE*	T-203G	rs405509	TT	TG	GG	2213	1847	451	8	0.491	0.409	0.100	0.483	0.424	0.093	0.025	0.305
*APOE*	Arg158Cys (C/T) (at exon2)	rs7412	CC	CT	TT	4140	369	9	1	0.916	0.082	0.002	0.916	0.082	0.002	0.72	0.043
*APOE*	Cys112Arg (T/C) (at exon4)	rs429358	TT	TC	CC	3669	782	67	1	0.812	0.173	0.015	0.808	0.182	0.010	0.001	0.101
*ARNTL(BMAL1)*	A/G	rs7950226	AA	AG	GG	1638	2102	773	6	0.363	0.466	0.171	0.355	0.482	0.163	0.028	0.404
*ART4(DOK1)*	Leu208Leu (G/A)	rs3088189	GG	GA	AA	3595	875	47	2	0.796	0.194	0.010	0.797	0.192	0.012	0.48	0.107
*ART4(DOK1)*	Asp265Asn (G/A)	rs11276	GG	GA	AA	3597	872	48	2	0.796	0.193	0.011	0.797	0.191	0.011	0.59	0.107
*BHMT*	Arg239Gln (G742A)	rs3733890	GG	GA	AA	2765	1511	241	2	0.612	0.335	0.053	0.607	0.344	0.049	0.069	0.221
*CBS*	Tyr233Tyr (C699T)	rs234706	CC	CT	TT	4302	212	2	3	0.953	0.047	0.000	0.953	0.047	0.001	1.00	0.024
*CD14*	T-260C/T-159C	rs2569190	TT	TC	CC	1304	2256	958	1	0.289	0.499	0.212	0.290	0.497	0.213	0.79	0.462
*CDKAL1*	G/C	rs7754840	GG	GC	CC	1581	2195	740	3	0.350	0.486	0.164	0.352	0.483	0.166	0.64	0.407
*CDKN2A/B*	T/C	rs10811661	TT	TC	CC	1379	2205	934	1	0.305	0.488	0.207	0.302	0.495	0.203	0.34	0.451
*CETP*	A-629C	rs1800775	AA	AC	CC	1384	2242	891	2	0.306	0.496	0.197	0.308	0.494	0.198	0.76	0.445
*CETP*	Ile405Val (A/G)	rs5882	AA	AG	GG	1236	2116	1166	1	0.274	0.468	0.258	0.258	0.500	0.242	<0.0001	0.492
*CETP*	TaqIB (C/T)	rs708272	CC	CT	TT	1603	2174	741	1	0.355	0.481	0.164	0.354	0.482	0.164	0.93	0.405
*CETP*	G/T	rs3764261	GG	GT	TT	2707	1512	298	2	0.599	0.335	0.066	0.588	0.358	0.054	<0.0001	0.233
*CHRNB2*	G-42A	rs2072658	GG	GA	AA	2837	1518	161	3	0.628	0.336	0.036	0.634	0.324	0.042	0.015	0.204
*CHRNB2*	C/T (at 3′UTR)	rs2072660	CC	CT	TT	2601	1641	275	2	0.576	0.363	0.061	0.574	0.367	0.059	0.44	0.243
*COMT*	Val158Met (G/A)	rs4680	GG	GA	AA	2006	1983	528	2	0.444	0.439	0.117	0.440	0.446	0.113	0.27	0.336
*CYP1A1*	Ile462Val (A/G)	rs1048943	AA	AG	GG	2630	1620	268	1	0.582	0.359	0.059	0.580	0.363	0.057	0.39	0.239
*CYP1A2*	A734C	rs762551	AA	AC	CC	1878	2043	592	6	0.416	0.453	0.131	0.413	0.459	0.128	0.33	0.358
*CYP11B2*	T-344C	rs1799998	TT	TC	CC	2031	1976	507	5	0.450	0.438	0.112	0.447	0.443	0.110	0.42	0.331
*CYP17A1*	T-34C	rs743572	TT	TC	CC	1281	2257	977	4	0.284	0.500	0.216	0.285	0.498	0.217	0.79	0.466
*ESR1*	IVS1-351A/G (*Xba* I)	rs11155816	AA	AG	GG	4515	0	0	4	1.000	0.000	0.000	1.000	0.000	0.000	1.00	0.000
*ESR1*	IVS1-397T/C (*Pvu* II)	rs2234693	TT	TC	CC	1508	2172	834	5	0.334	0.481	0.185	0.330	0.489	0.181	0.30	0.425
*ESR2*	Val328Val (G1082A) (*Rsa* I)	rs1256049	GG	GA	AA	2320	1823	373	3	0.514	0.404	0.083	0.512	0.407	0.081	0.58	0.284
*FTO*	T/A	rs9939609	TT	TA	AA	2881	1454	183	1	0.638	0.322	0.041	0.638	0.322	0.041	1.00	0.201
*GCK*	G-30A	rs1799884	GG	GA	AA	2989	1348	181	1	0.662	0.298	0.040	0.657	0.307	0.036	0.065	0.189
*GCKR*	A/G	rs780094	AA	AG	GG	1379	2221	917	2	0.305	0.492	0.203	0.304	0.495	0.201	0.67	0.449
*GCKR*	Leu446Pro (T/C)	rs1260326	TT	TC	CC	1402	2214	902	1	0.310	0.490	0.200	0.308	0.494	0.198	0.61	0.445
*GNAS1*	T393C	rs7121	TT	TC	CC	1466	2233	817	3	0.325	0.494	0.181	0.327	0.490	0.183	0.52	0.428
*IGF2BP2*	G/T (at intron)	rs4402960	GG	GT	TT	2215	1866	436	2	0.490	0.413	0.097	0.486	0.422	0.092	0.14	0.303
*IL-1B*	T-31C	rs1143627	TT	TC	CC	1331	2196	990	2	0.295	0.486	0.219	0.289	0.497	0.214	0.14	0.462
*IL-2*	T-330G	rs2069762	TT	TG	GG	2033	1986	494	6	0.450	0.440	0.109	0.450	0.442	0.109	0.79	0.329
*IL-4*	T-33C	rs2070874	TT	TC	CC	1997	1989	531	2	0.442	0.440	0.118	0.439	0.447	0.114	0.30	0.338
*IL-6*	C-634G	rs1800796	CC	CG	GG	2611	1607	298	3	0.578	0.356	0.066	0.572	0.369	0.059	0.019	0.244
*IL-8*	T-251A	rs4073	TT	TA	AA	2077	1952	463	27	0.462	0.435	0.103	0.462	0.435	0.103	0.89	0.320
*IL-10*	T-819C	rs1800871	TT	TC	CC	1942	2009	557	11	0.431	0.446	0.124	0.427	0.453	0.120	0.29	0.346
*IL-13*	C-1111T	rs1800925	CC	CT	TT	3009	1359	147	4	0.666	0.301	0.033	0.667	0.299	0.034	0.69	0.183
*KCNJ11*	Glu23Lys (C/T)	rs5219	CC	CT	TT	1822	2081	615	1	0.403	0.461	0.136	0.401	0.464	0.134	0.59	0.366
*LCAT/SLC12A4*	Ser232Thr (T/A)	rs4986970	TT	TA	AA	4516	0	0	3	1.000	0.000	0.000	1.000	0.000	0.000	1.00	0.000
*LIPC*	T-514C	rs1800588	TT	TC	CC	1178	2255	1085	1	0.261	0.499	0.240	0.260	0.500	0.240	0.93	0.490
*LIPC*	Val95Met (G/A)	rs6078	GG	GA	AA	2659	1606	252	2	0.589	0.356	0.056	0.587	0.358	0.055	0.65	0.234
*MPO*	G-463A	rs2333227	GG	GA	AA	3612	852	52	3	0.800	0.189	0.012	0.800	0.189	0.011	0.81	0.106
*MTHFD1*	Arg134Lys (C401T)	rs1950902	CC	CT	TT	2773	1517	228	1	0.614	0.336	0.050	0.611	0.341	0.048	0.28	0.218
*MTHFD1*	Arg653Gln (G1958A)	rs2236225	GG	GA	AA	2340	1790	384	5	0.518	0.397	0.085	0.514	0.406	0.080	0.12	0.283
*MTHFR*	Ala222Val (C677T)	rs1801133	CC	CT	TT	1763	2060	694	2	0.390	0.456	0.154	0.382	0.472	0.146	0.023	0.382
*MTHFR*	Glu429Ala (A1298C)	rs1801131	AA	AC	CC	3023	1323	169	4	0.670	0.293	0.037	0.666	0.300	0.034	0.11	0.184
*MTR*	Asp919Gly (A/G)	rs1805087	AA	AG	GG	2883	1446	185	5	0.639	0.320	0.041	0.638	0.321	0.040	0.82	0.201
*MTRR*	Ile22Met (A66G)	rs1801394	AA	AG	GG	2155	1925	436	3	0.477	0.426	0.097	0.477	0.428	0.096	0.83	0.310
*NOS3*	T-786C	rs2070744	TT	TC	CC	3602	863	48	6	0.798	0.191	0.011	0.799	0.190	0.011	0.70	0.106
*PPARD*	T-842C (at exon3)	rs2267668	TT	TC	CC	2922	1421	172	4	0.647	0.315	0.038	0.647	0.315	0.038	1.00	0.195
*PPARD*	T-48444C (at exon3)	rs6902123	TT	TC	CC	4343	173	2	1	0.961	0.038	0.000	0.961	0.038	0.000	0.69	0.020
*PPARD*	Asn163Asn (A65G) (at exon7)	rs2076167	AA	AG	GG	2780	1530	204	5	0.616	0.339	0.045	0.617	0.337	0.046	0.76	0.215
*PPARG*	Pro12Ala (C/G)	rs1801282	CC	CG	GG	4236	274	5	4	0.938	0.061	0.001	0.938	0.061	0.001	0.80	0.031
*PPARG*	His477His (C161T)	rs3856806	CC	CT	TT	3231	1198	86	4	0.716	0.265	0.019	0.720	0.257	0.023	0.043	0.152
*PPARGC1A*	Thr394Thr (C/T)	rs2970847	CC	CT	TT	2755	1539	221	4	0.610	0.341	0.049	0.609	0.343	0.048	0.76	0.219
*PPARGC1A*	Gly482Ser (G/A)	rs8192678	GG	GA	AA	1317	2247	952	3	0.292	0.498	0.211	0.292	0.497	0.211	0.93	0.460
*PRKAA2*	IVS4+961T/C	rs1418442	TT	TC	CC	2677	1581	251	10	0.594	0.351	0.056	0.591	0.355	0.053	0.38	0.231
*PRKAA2*	IVS7+81T/C	rs932447	TT	TC	CC	2679	1585	252	3	0.593	0.351	0.056	0.591	0.356	0.053	0.38	0.231
*PRKAA2*	A/T (at 3′UTR)	rs1342382	AA	AT	TT	2739	1556	218	6	0.607	0.345	0.048	0.607	0.344	0.049	0.90	0.221
*PTGS2(COX2)*	G-765C	rs20417	GG	GC	CC	4260	247	6	6	0.944	0.055	0.001	0.943	0.056	0.001	0.27	0.029
*PTGS2(COX2)*	C-163G	rs5270	CC	CG	GG	4379	136	3	1	0.969	0.030	0.001	0.969	0.031	0.000	0.10	0.016
*PTPN11*	G33861A	rs2301756	GG	GA	AA	2926	1418	174	1	0.648	0.314	0.039	0.647	0.314	0.038	0.89	0.195
*RETN*	C-420G	rs1862513	CC	CG	GG	1956	1977	585	1	0.433	0.438	0.129	0.425	0.454	0.121	0.015	0.348
*SCARB1*	Val135Ile (G/A)	rs5891	GG	GA	AA	4518	0	0	1	1.000	0.000	0.000	1.000	0.000	0.000	1.00	0.000
*SCARB1*	Ala350Ala (C1119T)	rs5888	CC	CT	TT	2678	1619	221	1	0.593	0.358	0.049	0.596	0.352	0.052	0.25	0.228
*SCARB1*	G/A (at intron)	rs3782287	GG	GA	AA	2676	1574	268	1	0.592	0.348	0.059	0.588	0.358	0.055	0.074	0.234
*SERPINC1*	C/G	rs677	CC	CG	GG	2762	1527	227	3	0.612	0.338	0.050	0.609	0.342	0.048	0.41	0.219
*SHMT1*	Leu435Phe (C1420T)	rs1979277	CC	CT	TT	3755	722	41	1	0.831	0.160	0.009	0.830	0.162	0.008	0.36	0.089
*SLC19A(RFC1)*	His27Arg (A80G)	rs1051266	AA	AG	GG	1536	2187	793	3	0.340	0.484	0.176	0.339	0.486	0.175	0.76	0.418
*SLC30A8*	Arg325Trp (C/T)	rs13266634	CC	CT	TT	1543	1992	976	8	0.342	0.442	0.216	0.317	0.492	0.191	<0.0001	0.437
*SRD5A2*	Leu89Val (C/G)	rs523349	CC	CG	GG	1334	2286	896	3	0.295	0.506	0.198	0.301	0.495	0.204	0.15	0.452
*TAS2R38*	Pro49Ala (C/G)	rs713598	CC	CG	GG	1399	2219	898	3	0.310	0.491	0.199	0.309	0.494	0.198	0.74	0.445
*TAS2R38*	Ala262Val (C/T)	rs1726866	CC	CT	TT	1401	2218	899	1	0.310	0.491	0.199	0.309	0.494	0.198	0.70	0.444
*TAS2R38*	Val296Ile (C/T)	rs10246939	CC	CT	TT	1401	2218	898	2	0.310	0.491	0.199	0.309	0.494	0.197	0.72	0.444
*TCF7L2*	C/T (at intron)	rs7903146	CC	CT	TT	4174	333	11	1	0.924	0.074	0.002	0.923	0.075	0.002	0.11	0.039
*TNF-A*	T-1031C	rs1799964	TT	TC	CC	3147	1244	127	1	0.697	0.275	0.028	0.696	0.277	0.027	0.75	0.166
*TNF-A*	C-857T	rs1799724	CC	CT	TT	2990	1365	162	2	0.662	0.302	0.036	0.661	0.304	0.035	0.70	0.187
*USF1*	C7131T	rs3737787	CC	CT	TT	2793	1506	218	2	0.618	0.333	0.048	0.616	0.338	0.046	0.43	0.215
*VDR*	Met1Thr (*Fok* I) (C/T)	rs2228570	CC	CT	TT	1768	2105	643	3	0.391	0.466	0.142	0.390	0.469	0.141	0.68	0.375

Abbreviations: HWE, Hardy–Weinberg equilibrium; MAF, minor allele frequency; SNP, single nucleotide polymorphism.

^a^AA, Aa, aa, and XX indicate homozygotes of major alleles, heterozygotes, homozygotes of minor alleles, and samples for which the genotype could not be determined, respectively.

^b^Based on the Hardy–Weinberg equilibrium.

The *P* value for departures from the Hardy–Weinberg equilibrium was less than 0.05 for 19 polymorphisms. However, the only genotypes for which the difference between the observed and expected frequencies exceeded 3% were the *CETP* Ile405Val (A/G) heterozygote and the *SLC30A8* Arg325Trp (C/T) heterozygote. As shown in Table 5, some polymorphisms demonstrated a considerable difference in MAF among the participating cohorts; for 32 of the 108 polymorphisms, including *ABCC11* Arg180Gly (T/C), there was a highly significant difference in MAF among study areas (*P* < 0.001).

**Table 5.** Minor allele frequencies of 107 selected genetic polymorphisms (106 SNPs and 1 insertion/deletion polymorphism) by study area

Gene	Polymorphism	rs number	Minor allele frequency by study area											*P*^a^

			Total	Chiba	Shizuoka	Okazaki	ACC	Takashima	Kyoto	Tokushima	Fukuoka	Saga	Amami	
*ABCA1*	C-565T	rs2422493	0.402	0.371	0.429	0.406	0.426	0.401	0.394	0.468	0.404	0.405	0.355	0.005
*ABCA1*	G-191C	rs1800976	0.402	0.370	0.429	0.406	0.425	0.403	0.403	0.468	0.408	0.405	0.356	0.006
*ABCA1*	G-17C	rs2740483	0.301	0.363	0.296	0.300	0.271	0.294	0.300	0.247	0.313	0.313	0.271	0.0002
*ABCA1*	Val825Ile (G/A)	rs2066715	0.356	0.354	0.358	0.331	0.354	0.350	0.347	0.353	0.360	0.358	0.389	0.51
*ABCA1*	Val771Met (G/A)	rs2066718	0.066	0.067	0.060	0.067	0.059	0.061	0.034	0.047	0.073	0.068	0.088	0.041
*ABCA1*	Arg1587Lys (G/A)	rs2230808	0.391	0.393	0.391	0.384	0.392	0.394	0.347	0.326	0.369	0.388	0.441	0.027
*ACE*	Insertion/Deletion (I/D)	rs1799752	0.365	0.373	0.369	0.346	0.363	0.365	0.313	0.353	0.357	0.341	0.425	0.003
*ADD1*	Trp460Gly (T/G)	rs4961	0.451	0.454	0.436	0.424	0.455	0.430	0.463	0.463	0.429	0.464	0.509	0.006
*ADH1B*	His47Arg (A/G)	rs1229984	0.239	0.220	0.232	0.200	0.247	0.220	0.250	0.184	0.266	0.223	0.318	<0.0001
*ADH1C*	Arg272Gln (C/T)	rs1693482	0.075	0.053	0.068	0.054	0.178	0.056	0.053	0.058	0.065	0.053	0.072	<0.0001
*ADIPOQ*	C-11377G	rs266729	0.251	0.245	0.239	0.254	0.238	0.252	0.300	0.279	0.257	0.250	0.255	0.60
*ADIPOQ*	Gly15Gly (T/G)	rs2241766	0.290	0.285	0.304	0.285	0.309	0.301	0.316	0.295	0.287	0.284	0.251	0.18
*ADIPOQ*	G276T	rs1501299	0.262	0.290	0.284	0.282	0.147	0.266	0.263	0.289	0.286	0.308	0.236	<0.0001
*ADIPOQ*	IVS-3971A/G	rs822396	0.061	0.053	0.049	0.069	0.057	0.079	0.050	0.047	0.073	0.057	0.056	0.063
*ADIPOR1*	G-8503A	rs6666089	0.032	0.029	0.040	0.034	0.028	0.027	0.044	0.026	0.029	0.024	0.040	0.29
*ADIPOR1*	C5843T	rs1342387	0.483	0.484	0.473	0.476	0.481	0.480	0.478	0.426	0.495	0.483	0.504	0.78
*ADIPOR1*	C10224G	rs7539542	0.216	0.226	0.230	0.201	0.202	0.223	0.219	0.263	0.233	0.215	0.194	0.21
*ADRB2*	Gln27Glu (C/G)	rs1042714	0.065	0.073	0.051	0.061	0.066	0.093	0.081	0.079	0.072	0.063	0.039	0.0001
*ADRB3*	Trp64Arg (T/C)	rs4994	0.195	0.184	0.173	0.195	0.184	0.195	0.147	0.168	0.193	0.218	0.240	0.001
*AGT*	Thr174Met (C/T)	rs4762	0.105	0.104	0.106	0.101	0.106	0.092	0.094	0.095	0.123	0.105	0.115	0.68
*AGT*	Thr235Met (C/T)	rs699	0.187	0.181	0.183	0.208	0.189	0.195	0.219	0.211	0.173	0.178	0.170	0.31
*AGTR1(ATR1)*	A1166C (at 3′UTR)	rs5186	0.087	0.089	0.093	0.083	0.088	0.076	0.081	0.089	0.080	0.082	0.104	0.61
*ALDH2*	Glu487Lys (G/A)	rs671	0.254	0.232	0.274	0.316	0.298	0.254	0.226	0.300	0.269	0.267	0.112	<0.0001
*APOA1*	Ala61Thr (C/T)	rs12718465	0.055	0.051	0.049	0.056	0.061	0.055	0.088	0.016	0.046	0.043	0.079	0.0005
*APOA1*	Arg184Pro (G/C)	rs5078	0.000	0.000	0.000	0.000	0.000	0.000	0.000	0.000	0.000	0.000	0.000	
*APOA5*	T-1131C	rs662799	0.345	0.355	0.336	0.322	0.337	0.326	0.338	0.342	0.342	0.337	0.412	0.002
*APOA5*	Gly185Cys (G553T)	rs2075291	0.069	0.084	0.062	0.057	0.073	0.068	0.063	0.074	0.064	0.079	0.065	0.36
*APOE*	T-203G	rs405509	0.305	0.289	0.297	0.294	0.302	0.256	0.306	0.295	0.318	0.308	0.383	<0.0001
*APOE*	Arg158Cys (C/T) (at exon2)	rs7412	0.043	0.045	0.048	0.048	0.035	0.042	0.050	0.042	0.044	0.048	0.030	0.49
*APOE*	Cys112Arg (T/C) (at exon4)	rs429358	0.101	0.105	0.105	0.101	0.123	0.101	0.078	0.068	0.109	0.102	0.075	0.026
*ARNTL(BMAL1)*	A/G	rs7950226	0.404	0.424	0.405	0.420	0.395	0.402	0.391	0.347	0.427	0.431	0.344	0.002
*ART4(DOK1)*	Leu208Leu (G/A)	rs3088189	0.107	0.114	0.114	0.130	0.084	0.113	0.078	0.068	0.105	0.108	0.109	0.022
*ART4(DOK1)*	Asp265Asn (G/A)	rs11276	0.107	0.114	0.113	0.130	0.084	0.113	0.078	0.068	0.105	0.108	0.109	0.023
*BHMT*	Arg239Gln (G742A)	rs3733890	0.221	0.235	0.236	0.241	0.229	0.203	0.209	0.226	0.243	0.217	0.164	0.0005
*CBS*	Tyr233Tyr (C699T)	rs234706	0.024	0.027	0.033	0.026	0.020	0.021	0.028	0.011	0.034	0.017	0.017	0.073
*CD14*	T-260C/T-159C	rs2569190	0.462	0.460	0.467	0.467	0.439	0.499	0.428	0.395	0.468	0.448	0.471	0.088
*CDKAL1*	G/C	rs7754840	0.407	0.428	0.390	0.430	0.392	0.411	0.363	0.374	0.424	0.435	0.367	0.009
*CDKN2A/B*	T/C	rs10811661	0.451	0.465	0.450	0.466	0.446	0.470	0.453	0.411	0.441	0.452	0.419	0.42
*CETP*	A-629C	rs1800775	0.445	0.445	0.463	0.442	0.436	0.441	0.469	0.405	0.462	0.422	0.458	0.52
*CETP*	Ile405Val (A/G)	rs5882	0.492	0.537	0.376	0.452	0.523	0.498	0.509	0.458	0.495	0.556	0.506	<0.0001
*CETP*	TaqIB (C/T)	rs708272	0.405	0.404	0.401	0.419	0.419	0.384	0.375	0.363	0.378	0.443	0.398	0.067
*CETP*	G/T	rs3764261	0.233	0.183	0.340	0.295	0.203	0.188	0.209	0.158	0.204	0.223	0.240	<0.0001
*CHRNB2*	G-42A	rs2072658	0.204	0.236	0.214	0.185	0.215	0.222	0.203	0.253	0.202	0.203	0.140	<0.0001
*CHRNB2*	C/T (at 3′UTR)	rs2072660	0.243	0.258	0.225	0.264	0.224	0.241	0.216	0.237	0.214	0.233	0.292	0.001
*COMT*	Val158Met (G/A)	rs4680	0.336	0.310	0.328	0.336	0.323	0.359	0.303	0.295	0.336	0.315	0.406	<0.0001
*CYP1A1*	Ile462Val (A/G)	rs1048943	0.239	0.222	0.235	0.256	0.222	0.244	0.250	0.268	0.222	0.225	0.274	0.069
*CYP1A2*	A734C	rs762551	0.358	0.336	0.349	0.356	0.344	0.367	0.352	0.384	0.351	0.363	0.391	0.38
*CYP11B2*	T-344C	rs1799998	0.331	0.334	0.318	0.339	0.289	0.294	0.346	0.284	0.329	0.325	0.434	<0.0001
*CYP17A1*	T-34C	rs743572	0.466	0.453	0.460	0.449	0.485	0.492	0.500	0.353	0.467	0.452	0.482	0.018
*ESR1*	IVS1-351A/G (*Xba* I)	rs11155816	0.000	0.000	0.000	0.000	0.000	0.000	0.000	0.000	0.000	0.000	0.000	
*ESR1*	IVS1-397T/C (*Pvu* II)	rs2234693	0.425	0.436	0.422	0.422	0.416	0.452	0.334	0.421	0.430	0.438	0.415	0.062
*ESR2*	Val328Val (G1082A) (*Rsa* I)	rs1256049	0.284	0.268	0.281	0.297	0.272	0.308	0.272	0.284	0.314	0.293	0.250	0.054
*FTO*	T/A	rs9939609	0.201	0.172	0.195	0.220	0.190	0.188	0.169	0.158	0.206	0.186	0.277	<0.0001
*GCK*	G-30A	rs1799884	0.189	0.193	0.179	0.183	0.186	0.182	0.138	0.147	0.304	0.173	0.159	<0.0001
*GCKR*	A/G	rs780094	0.449	0.428	0.436	0.440	0.439	0.425	0.350	0.453	0.450	0.445	0.562	<0.0001
*GCKR*	Leu446Pro (T/C)	rs1260326	0.445	0.430	0.434	0.439	0.438	0.416	0.353	0.421	0.447	0.432	0.559	<0.0001
*GNAS1*	T393C	rs7121	0.428	0.448	0.427	0.408	0.463	0.422	0.469	0.342	0.454	0.418	0.391	0.002
*IGF2BP2*	G/T (at intron)	rs4402960	0.303	0.328	0.306	0.292	0.298	0.296	0.309	0.268	0.300	0.304	0.306	0.81
*IL-1B*	T-31C	rs1143627	0.462	0.444	0.463	0.477	0.439	0.454	0.491	0.484	0.477	0.462	0.475	0.52
*IL-2*	T-330G	rs2069762	0.329	0.330	0.336	0.316	0.311	0.323	0.309	0.305	0.350	0.321	0.365	0.21
*IL-4*	T-33C	rs2070874	0.338	0.317	0.337	0.315	0.333	0.291	0.313	0.284	0.336	0.337	0.457	<0.0001
*IL-6*	C-634G	rs1800796	0.244	0.247	0.206	0.250	0.234	0.226	0.225	0.232	0.223	0.240	0.338	<0.0001
*IL-8*	T-251A	rs4073	0.320	0.334	0.321	0.318	0.320	0.304	0.331	0.266	0.339	0.329	0.306	0.56
*IL-10*	T-819C	rs1800871	0.346	0.329	0.346	0.320	0.340	0.313	0.356	0.268	0.354	0.359	0.425	<0.0001
*IL-13*	C-1111T	rs1800925	0.183	0.182	0.178	0.197	0.185	0.178	0.213	0.158	0.172	0.190	0.176	0.73
*KCNJ11*	Glu23Lys (C/T)	rs5219	0.366	0.386	0.364	0.382	0.343	0.374	0.363	0.316	0.361	0.366	0.368	0.52
*LCAT/SLC12A4*	Ser232Thr (T/A)	rs4986970	0.000	0.000	0.000	0.000	0.000	0.000	0.000	0.000	0.000	0.000	0.000	
*LIPC*	T-514C	rs1800588	0.490	0.522	0.478	0.496	0.484	0.497	0.522	0.484	0.502	0.503	0.429	0.006
*LIPC*	Val95Met (G/A)	rs6078	0.234	0.232	0.229	0.221	0.269	0.259	0.247	0.237	0.208	0.253	0.182	<0.0001
*MPO*	G-463A	rs2333227	0.106	0.107	0.105	0.107	0.102	0.113	0.100	0.100	0.106	0.093	0.119	0.84
*MTHFD1*	Arg134Lys (C401T)	rs1950902	0.218	0.214	0.216	0.218	0.212	0.229	0.184	0.195	0.217	0.239	0.215	0.66
*MTHFD1*	Arg653Gln (G1958A)	rs2236225	0.283	0.271	0.273	0.269	0.265	0.280	0.284	0.279	0.278	0.301	0.331	0.036
*MTHFR*	Ala222Val (C677T)	rs1801133	0.382	0.387	0.402	0.388	0.411	0.393	0.375	0.442	0.410	0.364	0.288	<0.0001
*MTHFR*	Glu429Ala (A1298C)	rs1801131	0.184	0.195	0.189	0.202	0.193	0.193	0.225	0.191	0.147	0.222	0.105	<0.0001
*MTR*	Asp919Gly (A/G)	rs1805087	0.201	0.188	0.210	0.186	0.183	0.179	0.184	0.232	0.193	0.176	0.299	<0.0001
*MTRR*	Ile22Met (A66G)	rs1801394	0.310	0.292	0.314	0.298	0.315	0.296	0.306	0.337	0.318	0.297	0.346	0.24
*NOS3*	T-786C	rs2070744	0.106	0.111	0.125	0.117	0.112	0.098	0.119	0.122	0.109	0.102	0.068	0.004
*PPARD*	T-842C (at exon3)	rs2267668	0.195	0.175	0.199	0.201	0.183	0.205	0.213	0.221	0.192	0.190	0.210	0.55
*PPARD*	T-48444C (at exon3)	rs6902123	0.020	0.015	0.020	0.030	0.025	0.017	0.019	0.016	0.019	0.019	0.012	0.15
*PPARD*	Asn163Asn (A65G) (at exon7)	rs2076167	0.215	0.190	0.212	0.231	0.206	0.221	0.225	0.234	0.209	0.205	0.236	0.30
*PPARG*	Pro12Ala (C/G)	rs1801282	0.031	0.031	0.027	0.028	0.037	0.047	0.022	0.011	0.029	0.032	0.025	0.064
*PPARG*	His477His (C161T)	rs3856806	0.152	0.153	0.159	0.143	0.149	0.167	0.159	0.147	0.177	0.166	0.099	0.0002
*PPARGC1A*	Thr394Thr (C/T)	rs2970847	0.219	0.229	0.223	0.237	0.219	0.222	0.209	0.237	0.208	0.243	0.168	0.005
*PPARGC1A*	Gly482Ser (G/A)	rs8192678	0.460	0.472	0.447	0.444	0.431	0.448	0.463	0.442	0.471	0.438	0.538	<0.0001
*PRKAA2*	IVS4+961T/C	rs1418442	0.231	0.216	0.228	0.232	0.206	0.217	0.184	0.237	0.205	0.237	0.318	<0.0001
*PRKAA2*	IVS7+81T/C	rs932447	0.231	0.216	0.228	0.232	0.207	0.218	0.184	0.237	0.205	0.239	0.318	<0.0001
*PRKAA2*	A/T (at 3′UTR)	rs1342382	0.221	0.238	0.220	0.200	0.250	0.225	0.204	0.211	0.233	0.197	0.212	0.072
*PTGS2(COX2)*	G-765C	rs20417	0.029	0.028	0.033	0.031	0.033	0.027	0.025	0.053	0.026	0.030	0.017	0.22
*PTGS2(COX2)*	C-163G	rs5270	0.016	0.017	0.023	0.017	0.020	0.015	0.009	0.011	0.009	0.011	0.016	0.32
*PTPN11*	G33861A	rs2301756	0.195	0.194	0.199	0.172	0.167	0.185	0.184	0.205	0.173	0.159	0.320	<0.0001
*RETN*	C-420G	rs1862513	0.348	0.351	0.328	0.330	0.343	0.330	0.334	0.316	0.340	0.366	0.411	0.002
*SCARB1*	Val135Ile (G/A)	rs5891	0.000	0.000	0.000	0.000	0.000	0.000	0.000	0.000	0.000	0.000	0.000	
*SCARB1*	Ala350Ala (C1119T)	rs5888	0.228	0.240	0.212	0.226	0.228	0.225	0.253	0.205	0.240	0.240	0.213	0.59
*SCARB1*	G/A (at intron)	rs3782287	0.234	0.225	0.223	0.243	0.227	0.249	0.297	0.189	0.207	0.219	0.263	0.008
*SERPINC1*	C/G	rs677	0.219	0.220	0.197	0.209	0.238	0.223	0.238	0.211	0.220	0.227	0.217	0.58
*SHMT1*	Leu435Phe (C1420T)	rs1979277	0.089	0.072	0.089	0.071	0.093	0.092	0.084	0.095	0.084	0.091	0.117	0.026
*SLC19A(RFC1)*	His27Arg (A80G)	rs1051266	0.418	0.415	0.435	0.412	0.426	0.423	0.397	0.395	0.430	0.454	0.352	0.001
*SLC30A8*	Arg325Trp (C/T)	rs13266634	0.437	0.439	0.449	0.443	0.438	0.418	0.472	0.453	0.434	0.451	0.411	0.53
*SRD5A2*	Leu89Val (C/G)	rs523349	0.452	0.437	0.468	0.430	0.458	0.458	0.391	0.399	0.449	0.471	0.464	0.13
*TAS2R38*	Pro49Ala (C/G)	rs713598	0.445	0.448	0.439	0.419	0.427	0.454	0.434	0.479	0.445	0.435	0.491	0.080
*TAS2R38*	Ala262Val (C/T)	rs1726866	0.444	0.449	0.438	0.419	0.427	0.454	0.434	0.479	0.444	0.435	0.491	0.078
*TAS2R38*	Val296Ile (C/T)	rs10246939	0.444	0.448	0.438	0.419	0.427	0.454	0.434	0.479	0.444	0.435	0.491	0.079
*TCF7L2*	C/T (at intron)	rs7903146	0.039	0.051	0.039	0.042	0.043	0.037	0.034	0.042	0.025	0.034	0.043	0.27
*TNF-A*	T-1031C	rs1799964	0.166	0.175	0.148	0.165	0.157	0.153	0.153	0.237	0.151	0.180	0.188	0.024
*TNF-A*	C-857T	rs1799724	0.187	0.172	0.171	0.173	0.166	0.161	0.194	0.184	0.203	0.199	0.255	<0.0001
*USF1*	C7131T	rs3737787	0.215	0.229	0.223	0.220	0.234	0.217	0.263	0.216	0.196	0.204	0.178	0.020
*VDR*	Met1Thr (*Fok* I) (C/T)	rs2228570	0.375	0.344	0.393	0.368	0.380	0.381	0.391	0.437	0.402	0.377	0.343	0.048

Abbreviations: ACC, Aichi Cancer Center; SNP, single nucleotide polymorphism.

^a^*P* for difference among study areas.

The Figure shows a comparison of the allele frequencies in our study population and the HapMap-JPT data set. Among 88 polymorphisms, only 5 (*ABCA1* rs2230808, *COMT* rs4680, *IL-6* rs1800796, *NOS3* rs2070744, and *VDR* rs2228570) showed a difference in allele frequencies of more than 0.1 between the 2 populations.

**Figure.**
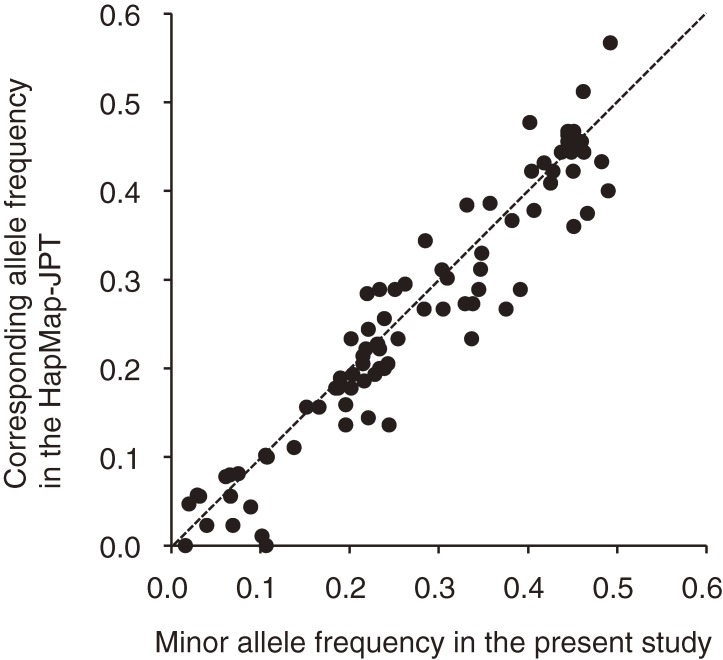
Scatter plot of allele frequencies for 88 polymorphisms in the data set from the Japan Multi-institutional Collaborative Cohort (J-MICC) Study and the HapMap-JPT data set registered at the US National Library of Medicine (http://www.ncbi.nlm.nih.gov/snp). Points on the identity (dotted) line represent allele frequencies that are identical in the 2 populations.

## DISCUSSION

The present report describes the profiles of participants in a cross-sectional study within the J-MICC Study data set and the allele frequencies of 108 polymorphisms, with potential relevance to lifestyle and clinical factors, in their genomes. The allele frequencies for most polymorphisms in our study population were comparable to those in the HapMap-JPT data set.

It has been suggested that polymorphisms for *APOA1* 184Pro (C), *ESR1* IVS1-351G, *LCAT/SLC12A4* 232Thr (A), and *SCARB1* 135Ile (A) do not exist in the Japanese population (http://www.ncbi.nlm.nih.gov/snp and personal communication); however, we included them in the present study to test this notion in a large sample (>4000 people). Our results confirmed that these minor alleles were indeed absent among Japanese.

Of the remaining 104 polymorphisms, 19 showed departures from the Hardy–Weinberg equilibrium with *P* values <0.05. In most cases, however, the absolute differences between the actual and expected frequencies were minimal. Thus, these apparently small *P* values could be accounted for by the large sample size and the multiple tests used in our study, and any errors in genotyping seemed unlikely to have resulted in substantial misclassification.

Although genotype data for only 45 people, at most, are available in the HapMap-JPT data set, the allele frequencies in the HapMap-JPT population and our study population were remarkably similar for most of the polymorphisms examined (Figure). For 45 individuals, the 95% confidence intervals were 0.047 to 0.181, 0.208 to 0.406, and 0.393 to 0.607 for MAF values of 0.1, 0.3, and 0.5, respectively, based on a binomial distribution.

A major strength of the current study was that it provided a comprehensive collection of data on lifestyle and clinical factors. Because it is not easy to gain access to data on genotype distributions in a large Japanese population, our data might also be useful as a reference tool. However, because the participants in this study were recruited from various sources throughout Japan, associations of genotypes with lifestyle and clinical factors might vary between populations. There might also have been differences between institutions in terms of the measurement methods used in the clinical examinations, because we could not directly control the quality of the health examinations. These differences must be taken into consideration when analyzing and interpreting the data. In addition, some polymorphisms showed a substantial difference in MAF among the participating cohorts (Table 5). Yamaguchi-Kabata et al suggested that individuals from the Ryukyu Islands, including the Amami Islands, had genetic characteristics that differed considerably from those of individuals from the main islands of Japan,^22^ which was consistent with our present results. Genetic variations among study areas should be taken into account in the data analysis. Furthermore, the generalizability of the study findings should be considered because the response rates were low in some areas. In most cases, however, the underlying biological mechanisms are unlikely to differ between the respondents and members of the general population. The low response rate might have been due to the recruitment methods (mailing invitation letters or distributing leaflets to the general populations of 3 areas) or the strict procedures used to obtain informed consent.

In conclusion, this comprehensive data collection on lifestyle and clinical factors will be useful in elucidating gene–environment interactions and could provide an informative reference tool, particularly because free access to genotype data for a large Japanese population is not readily available.
