# Profile of Participants and Genotype Distributions of 108 Polymorphisms in a Cross-Sectional Study of Associations of Genotypes With Lifestyle and Clinical Factors: A Project in the Japan Multi-Institutional Collaborative Cohort (J-MICC) Study

**DOI:** 10.2188/jea.JE20110072

**Published:** 2011-09-05

**Authors:** Kenji Wakai

In this article, we found errors in the genotype data for 5 single nucleotide polymorphisms (SNPs). We therefore corrected Tables 4 and 5 (only the revised data are presented). In addition, the main text from the last sentence of the third paragraph through the end of the fourth paragraph of the Results section should be amended as follows: “For the remaining 104 polymorphisms, the MAF varied from 0.016 (*PTGS2*(*COX2*) C-163G) to 0.479 (*CETP* Val405Ile (G/A)), and most of the variations were common (MAF ≥0.05 for 96 polymorphisms).

**Table 4.** Genotype distributions and allele frequencies of 108 selected genetic polymorphisms (corrected part only)

Gene	Polymorphism	rs number	Genotype^a^			*n*^a^				Frequency (proportion)						*P* for HWE	MAF

										Observed			Expected^b^				
			
			AA	Aa	aa	AA	Aa	aa	XX	AA	Aa	aa	AA	Aa	aa		
*ADH1C*	Arg272Gln (C/T)	rs1693482	CC	CT	TT	4027	474	15	3	0.892	0.105	0.003	0.892	0.105	0.003	0.78	0.056
*ADIPOQ*	G276T	rs1501299	GG	GT	TT	2353	1791	374	1	0.521	0.396	0.083	0.517	0.404	0.079	0.21	0.281
*CETP*	Val405Ile (G/A)	rs5882	GG	GA	AA	1254	2198	1066	1	0.278	0.486	0.236	0.271	0.499	0.230	0.089	0.479
*CETP*	G/T	rs3764261	GG	GT	TT	2877	1430	210	2	0.637	0.317	0.046	0.632	0.326	0.042	0.061	0.205
*GCK*	G-30A	rs1799884	GG	GA	AA	3056	1317	145	1	0.676	0.292	0.032	0.676	0.292	0.032	0.84	0.178

Abbreviations: HWE, Hardy-Weinberg equilibrium; MAF, minor allele frequency; SNP, single nucleotide polymorphism.

^a^AA, Aa, aa and XX indicate homozygotes of major alleles, heterozygotes, homozygotes of minor alleles, and samples of which genotype could not be determined, respectively.

^b^Based on the Hardy-Weinberg equilibrium.

**Table 5.** Minor allele frequencies of selected genetic polymorphisms by study area (corrected part only)

Gene	Polymorphism	rs number	Minor allele frequency by study area											*P*^a^

			Total	Chiba	Shizu- oka	Okazaki	ACC	Taka- shima	Kyoto	Toku- shima	Fukuoka	Saga	Amami	
*ADH1C*	Arg272Gln (C/T)	rs1693482	0.056	0.053	0.068	0.035	0.046	0.056	0.053	0.058	0.065	0.053	0.072	0.013
*ADIPOQ*	G276T	rs1501299	0.281	0.290	0.284	0.301	0.279	0.266	0.263	0.289	0.286	0.308	0.236	0.024
*CETP*	Val405Ile (G/A)	rs5882	0.479	0.463	0.478	0.462	0.477	0.502	0.491	0.542	0.505	0.444	0.494	0.058
*CETP*	G/T	rs3764261	0.205	0.183	0.194	0.209	0.203	0.188	0.209	0.158	0.204	0.223	0.240	0.028
*GCK*	G-30A	rs1799884	0.178	0.193	0.179	0.183	0.186	0.179	0.138	0.147	0.191	0.173	0.159	0.29

Abbreviations: ACC, Aichi Cancer Center; SNP, single nucleotide polymorphism.

^a^*P* for difference among study areas.

The *P* value for departures from the Hardy-Weinberg equilibrium was less than 0.05 for 15 polymorphisms. However, the only genotype for which the difference between the observed and expected frequencies exceeded 3% was the *SLC30A8* Arg325Trp (C/T) heterozygote. As shown in Table 5, some polymorphisms demonstrated a considerable difference in MAF among the participating cohorts; for 27 of the 108 polymorphisms, including *ABCC11* Arg180Gly (T/C), there was a highly significant difference in MAF among study areas (*P* < 0.001).”

Furthermore, the first sentence of the third paragraph of the Discussion should read: “Of the remaining 104 polymorphisms, 15 showed departures from the Hardy-Weinberg equilibrium, with *P* values less than 0.05.”

The authors regret these errors.

